# Mean Nocturnal Baseline Impedance (MNBI) Provides Evidence for Standardized Management Algorithms of Nonacid Gastroesophageal Reflux-Induced Chronic Cough

**DOI:** 10.1155/2023/7992062

**Published:** 2023-03-11

**Authors:** Yiqing Zhu, Tongyangzi Zhang, Shengyuan Wang, Wanzhen Li, Wenbo Shi, Xiao Bai, Bingxian Sha, Mengru Zhang, Siwan Wen, Cuiqin Shi, Xianghuai Xu, Li Yu

**Affiliations:** Department of Pulmonary and Critical Care Medicine, Tongji Hospital, School of Medicine, Tongji University, Shanghai 200065, China

## Abstract

**Background:**

The clinical management of nonacid gastroesophageal reflux-induced chronic cough (GERC) is challenging, and patient response to standard antireflux therapy (omeprazole 20 mg twice daily plus mosapride 10 mg thrice daily) is suboptimal. This study aimed to identify predictors of standard antireflux therapy efficacy and provide evidence for standardized management algorithms of nonacid GERC.

**Methods:**

A total of 115 nonacid GERC patients who underwent multichannel intraluminal impedance-pH monitoring (MII-pH) were enrolled between March 2017 and March 2021. Retrospective analysis of general information and MII-pH indications were used to establish a regression analysis model for multiple factors affecting standard antireflux therapy efficacy.

**Results:**

90 patients met the inclusion criteria, and the overall response rate to standard antireflux therapy was 55.5% (50/90). The mean nocturnal baseline impedance (MNBI) (1817.75 ± 259.26 vs. 2369.93 ± 326.35, *P* = 0.030) and proximal MNBI (1833.39 ± 92.16 vs. 2742.57 ± 204.64, *P* ≤ 0.001) of responders were lower than those of nonresponders. Weakly acid reflux (56.00 (31.70, 86.00) vs. 14.00 (14.00, 44.20), *P* = 0.022), nonacid reflux (61.35 (15.90.86.50) vs. 21.60 (0.00, 52.50), *P* = 0.008), and proximal extent (19.00 (5.04, 24.00) vs. 5.50 (2.56, 11.13), *P* = 0.011) were markedly higher in responders than nonresponders. Proximal MNBI (OR = 0.997, *P* = 0.042, and optimal cutoff = 2140 Ω) and weakly acid reflux (OR = 1.051, *P* = 0.029, and optimal cutoff = 45) were independent predictors of standard antireflux therapy efficacy. The combination predictive value did not show better results than either individual predictor.

**Conclusions:**

Proximal MNBI < 2140 Ω may be used to screen patients with nonacid GERC suitable for standard antireflux therapy and in standardized management algorithms for nonacid GERC. In the absence of MNBI, weakly acid reflux > 45 can be used as an auxiliary indicator.

## 1. Introduction

Gastroesophageal reflux-induced chronic cough (GERC), a specific type of gastroesophageal reflux (GER) disease, is one of the most common causes of chronic cough [1, 2]. Based on its pH value, GER can be divided into the following two major subtypes: acid and nonacid. These subtypes can be further divided into weakly acid and weakly alkaline reflux. Reflux with pH values ≤ 4, 4.1–7, and  ≥ 7 is considered acid, weakly acid, and weakly alkaline reflux, respectively [3]. Acid reflux is the main cause of GERC, which has led to a considerable amount of research into its diagnosis and treatment. However, nonacid reflux, which also plays an important role in GERC, lacks sufficient investigation [4]. The rate of nonacid GERC has been shown to be 37% and 80% in chronic cough patients without proton pump inhibitor (PPI) treatment and those with PPI treatment, respectively. 26% of chronic cough patients have a positive symptom index (SI), which indicates a nonacid-related cough [5]. The lack of multichannel intraluminal impedance-pH monitoring (MII-pH) has led to an underestimation of the diagnostic rate and accuracy of nonacid GERC.

The current first-line treatment for GERC is PPIs [6, 7]. For patients with acid exposure identified by MII-pH monitoring, 8 weeks of standard antireflux therapy, such as 20 mg omeprazole twice daily plus 10 mg mosapride thrice daily, is recommended. PPIs have been shown to be effective in GERC patients with definite acid exposure [6, 8, 9]. Therefore, PPIs have achieved great success in treating GERC, especially acid GERC, by reducing the acidity of regurgitates. Although PPIs are not generally recommended for patients with nonacid GERC [8], studies have shown that nonacid reflux is reduced or disappears in some patients receiving PPI therapy. Several previous studies have shown that standard antireflux therapy is effective in more than one-third of nonacid GERC patients [10–13]. For patients unresponsive to standard antireflux therapy, transient lower oesophageal sphincter relaxations (TLESRs) and oesophageal dysmotility might be the underlying aetiology. Baclofen has shown its antitussive efficacy in these patients by decreasing both acid and weakly acid reflux as well as the number of reflux episodes and reflux-related symptoms [3, 11, 14]. However, due to the prominent neurological side effects such as sedation, the application of baclofen was usually prudent. Patients had experienced at least 8 weeks of nonresponse to standard antireflux therapy before baclofen initiation, resulting in poor adherence in nonacid GERC cases.

Therefore, this study aimed to identify the potential factors related to the therapeutic efficacy of standard antireflux therapy in nonacid GERC patients. Retrospective analysis of the clinical data of nonacid GERC patients was used to identify nonacid GERC patients for whom standard antireflux therapy was suitable. For patients for whom the therapy was not suitable, baclofen may be administered without unnecessary delay to improve the efficiency and accuracy of treatment.

## 2. Methods

### 2.1. Patients

Patients with nonacid GERC as the sole explanation of chronic cough attending our respiratory clinic between March 2017 and March 2021 were recruited. After detailed medical history inquiries and physical examination as described previously [1], all patients underwent chest X-ray, lung function test, histamine bronchial provocation test [15], capsaicin cough sensitivity test [16], induced sputum cytological examination [17], and MII-pH [11] to identify the causes of chronic cough.

The diagnosis of nonacid GERC was based on previous studies [12, 13, 18, 19], the Lyon consensus [20], and Chinese cough guidelines [7]. Patients who met the following criteria were diagnosed with nonacid GERC: (1) Cough lasting for ≥ 8 weeks, with or without regurgitation or heartburn; (2) Results of MII-pH monitoring satisfied (1) and (2) simultaneously [11–13, 18, 21], where (1) was an AET of < 6% and (2) was an SAP for nonacid reflux ≥ 95% and/or an SI for nonacid reflux ≥ 50% and/or nonacid reflux > 80; and (3) Cough completely resolved or significantly improved (cough symptom score decreased by > 50%) in response to stepwise antireflux therapy [10]. Standard antireflux therapy (omeprazole 20 mg twice daily plus mosapride 10 mg thrice daily) was first introduced to nonacid GERC patients for 8 weeks. Patients who responded to standard antireflux therapy were kept on this treatment until their cough resolved; otherwise, baclofen was introduced (omeprazole 20 mg twice daily and baclofen initially at 10 mg thrice daily and gradually increased to 20 mg thrice daily) for additional 4 weeks. Nonacid GERC patients who responded to standard antireflux treatment were classified as the standard antireflux-responsive group; conversely, the other patients were classified as the standard antireflux-unresponsive group.

Cough severity was evaluated at the first visit and every 2 weeks during the follow-up period using the cough symptom score described by Hsu et al. [22]. The gastroesophageal reflux diagnostic questionnaire (GerdQ), described by Xu et al., was employed to evaluate patients' upper gastrointestinal symptoms [12]. Capsaicin cough sensitivity was evaluated according to the European Respiratory Society (ERS) guidelines [8] and using the modified method described by Fujimura et al. [16] with the lowest capsaicin inhalation concentration required to induce ≥2 (C2) or ≥5 (C5) coughs as the subject's cough threshold.

Patients who met all of the nonacid GERC criteria mentioned above were included in the study. Patients with other causes of chronic cough or incomplete medical records were excluded.

The study was approved by the Ethics Committee of Tongji Hospital (K-2020-025).

### 2.2. Laboratory Examination

Measurement of MII-pH [11, 23] was carried out by transnasal insertion of a 2.1 mm diameter combined MII-pH catheter consisting of six impedance channel amplifiers (K6011-E10632, Unisensor, Switzerland) and an antimony pH probe (819100, Medical Measurement System B.V., Netherlands) into the oesophagus. The MII-pH catheter was positioned with the pH electrode 5 cm above the lower oesophageal sphincter (LES) and the six impedance recording channels (*z*1, *z*2, *z*3, *z*4, *z*5, and *z*6) at 17, 15, 9, 7, 5, and 3 cm above the LES, respectively. A connected portable data logger (Ohmega; Medical Measurement System BV) was used to store data at 50 Hz frequency from all seven channels over 24 h. MII-pH measures changes in conductivity of an alternating electrical current with a pair of metallic rings mounted on a catheter. Electrical impedance was expressed in ohms (Ω) and was equivalent to the resistance to the direct electrical current. Baseline impedance (BI) [24] values reflected the status of the oesophageal mucosa. Event markers were used to record the occurrence of symptoms, times of meals, and changes in posture. Supporting professional analysis software was used for data analysis.

Before MII-pH monitoring, patients who had recently used acid suppressants, potassium chloride, or nonsteroidal anti-inflammatory drugs that may cause reflux were excluded. During the MII-pH monitoring period, patients were encouraged to perform their regular activities and have routine meals; they were given a personal diary to note the time of meal, sleep, recumbent position, and any cough-related symptoms. The following key indicators were recorded using the accompanying professional software (MMS database, v8.7) for analysis: ① acid exposure time (AET) defined as the total time (%) with pH <4 divided by the total time of monitoring; notably, the Lyon Consensus proposes that an AET < 4% is considered normal (physiological) and an AET > 6% is clinically abnormal, with intermediate values between these limits being inconclusive [20]; ② total reflux times, where > 80 reflux episodes per 24 hours were considered abnormal [20]; ③ the number of proximal reflux episodes (15 cm above the LES); ④ the oesophageal acid clearance time (the time required for pH to recover to ≥ 4.0 after acid reflux); ⑤ the symptom association probability (SAP) defined as the temporal association between cough recorded by patients in a diary and reflux that had occurred during the preceding 2 min period; ⑥ the symptom index (SI) defined as the percentage of reflux-related symptom episodes (cough) among the total number of symptom episodes; ⑦ the mean nocturnal baseline impedance (MNBI) [25] assessed from the most distal impedance channel during the nighttime recumbent period; three 10 min time periods (approximately 1.00 am, 2.00 am, and 3.00 am) were selected, and the mean was calculated, without periods of swallows and refluxes or pH drops; distal MNBI was calculated as the average of MNBI values from the channels located at 3, 5, 7, and 9 cm above the LES; proximal MNBI was calculated as the average of MNBI values from the channels located at 15 and 17 cm above the LES; and ⑧ postreflux swallow-induced peristaltic wave (PSPW) index [25], where PSPWs were defined as antegrade 50% drops in impedance, originating in the proximal oesophagus and reaching the distal lumen within 30 s after reflux events. The PSPW index was calculated by dividing the number of PSPWs by the number of reflux events. The MNBI and PSPW index were calculated by the same operator blinded to the treatment outcomes of each patient.

### 2.3. Review of Clinical Information

A retrospective analysis was performed to compare the general information, MII-pH monitoring parameters, and other assessments between the responsive and nonresponsive groups. Stepwise logistic regression was then performed to identify the indicators of the initial assessments of the therapeutic response to standard antireflux therapy in nonacid GERC patients.

### 2.4. Statistical Analysis

The normally distributed data are expressed as the mean ± standard deviation (SD), while skewed data are expressed as the median (interquartile range). After logarithmic transformation, C2 and C5 were expressed as the geometric mean ± SD. Gender and coughing properties were compared by the *χ*^2^ test. The analysis of variance and the Kruskal–Wallis rank sum test were used to compare the data between groups when applicable. Spearman and Pearson's tests were used for the correlation analysis. A univariate regression analysis was performed to screen significant variables, and then, a stepwise multiple logistic regression analysis was used to identify independent predictors for the therapeutic efficacy of standard antireflux therapy. Receiver operating characteristic (ROC) curves were plotted for the model equation and then compared using the DeLong test. SPSS 21.0 software (SPSS, Chicago, IL, USA) was applied for statistical calculation. A *P* value <0.05 was considered statistically significant.

## 3. Results

### 3.1. General Information

A total of 975 patients with complete information over the period studied were diagnosed with chronic cough. Of these, 273 were diagnosed with GERC. A total of 115 nonacid GERC patients met the inclusion criteria of this study, accounting for 42.1% of GERC patients in the same period. Four patients with incomplete follow-up data and twenty-one patients with dual/triple aetiologies were excluded. Of the 21 excluded patients, 6 had nonacid GERC combined with cough variant asthma (CVA), 5 had upper airway cough syndrome (UACS), 5 had eosinophilic bronchitis (EB), and 1 had obstructive sleep apnoea syndrome (OSAS). In addition, 4 patients had 3 aetiologies, including 2 patients with nonacid GERC plus UACS and CVA, 1 patient with nonacid GERC plus UACS and OSAS, and 1 patient with nonacid GERC plus UACS and EB. The remaining 90 out of the original 975 patients were included in the study, as they had only one aetiology (nonacid GERC).

In these 90 patients, 10% (*n* = 9) had heartburn, 23.3% (*n* = 21) regurgitation, 40% (*n* = 36) had belching, and 14.4% (*n* = 13) had coughing while eating (during or soon after meals). None of the patients had undergone previous fundoplication or foregut surgery. Eleven patients were examined with gastroduodenoscopy, of which two patients had oesophagitis and none had Barrett's oesophagus. According to the Chicago classification of oesophageal motility v3.0 [26], 14.4% (*n* = 13) of patients had major disorders of peristalsis, 41.2% (*n* = 37) had minor disorders of peristalsis, and 44.4% (*n* = 40) had normal oesophageal motility.

All recruited patients completed the standard antireflux treatment. Cough was controlled in 50 patients (55.5%); the remaining 40 patients (44.5%) responded to further baclofen initiation. A total of 40 patients were treated with baclofen in this study. The main side effects of baclofen were somnolence (32.5%, 13/40), dizziness (20%, 8/40), fatigue (27.5%, 11/40), and nausea (2.5%, 1/40), but most patients could tolerate the side effects, and they were gradually alleviated or disappeared within 3 weeks of treatment. There was no difference in demographic and clinical characteristics between the antireflux-responsive group and the antireflux-unresponsive group (Table 1).

### 3.2. Differences in MII-pH Data of Responders and Nonresponders

The MNBI was lower in responders than in nonresponders (1817.75 ± 259.26 vs. 2369.93 ± 326.35, *P*=0.030), and the proximal MNBI was lower in responders than in nonresponders (1833.39 ± 92.16 vs. 2742.57 ± 204.64, *P* ≤ 0.001). Markedly more weakly acid reflux (56.00 (31.70, 86.00) vs. 14.00 (14.00, 44.20), *P*=0.022), nonacid reflux (61.35 (15.90, 86.50) vs. 21.60 (0.00, 52.50), *P*=0.008), and proximal extent (19.00 (5.04, 24.00) vs. 5.50 (2.56, 11.13), *P*=0.011) were found in responders compared with nonresponders. Gas reflux in the responders tended to be higher than that in the nonresponders, although the difference was not statistically significant (20.00 (10.40, 44.00) vs. 11.10 (4.85, 24.00), *P*=0.059).

The other indices, including AET, SAP, SI, and total reflux times, which are important indicators for the diagnosis of GERC, in addition to the PSPW index, were not significantly different between the two groups (Table 2).

### 3.3. Factors Associated with the Therapeutic Efficacy of Standard Antireflux Therapy

The variables with statistically significant differences between the responders and nonresponders in Tables 1 and 2 were selected for the univariate logistic analysis. Among the significant factors identified by univariate logistic analysis, multivariate logistic regression revealed that proximal MNBI (OR = 0.997, *P*=0.042) and weak acid reflux (OR = 1.051, *P*=0.029) were independent predictors of standard antireflux therapy (Table 3).

Stepwise logistic regression resulted in a significant equation: Logit *P*=2.573 − 0.002*X*1 + 0.039*X*2, where *X*1 represents the score of “Proximal MNBI” and *X*2 represents the score of “Weakly acid reflux.” The equation explained 42.2% of the variation in the response rate to standard antireflux treatment (Nageire *R*2) and correctly screened 88.50% of subjects. The Hosmer–Lemeshow test (*χ*^2^ (2) = 1.103, *P*=0.993) verified its sufficient calibration.

**Figure 1:**
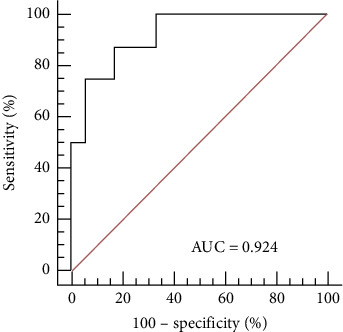
The internal accuracy of the logistic regression model assessed by ROC curve analysis. The AUC was 0.924 (95% CI: 0.749–0.9591; *P* ≤ 0.001), which indicates that for 92.4% of the paired participants (one responder, one nonresponder), the responder scored higher. These results suggest that the logistic regression model used in this study had a good ability to discriminate between responsive and unresponsive participants. AUC, area under the curve; CI, confidence interval; ROC, receiver operating characteristic.

When *P* ≥ 0.3772 was used as a cutoff point, the logistic regression equation effectively discriminated the responders from the nonresponders, with a sensitivity of 87.5% and a specificity of 83.3%, as indicated by the ROC analysis (Figure 1).

### 3.4. Proximal MNBI and Weak Acid Reflux for Predicting Standard Antireflux Therapy Efficacy

**Figure 2:**
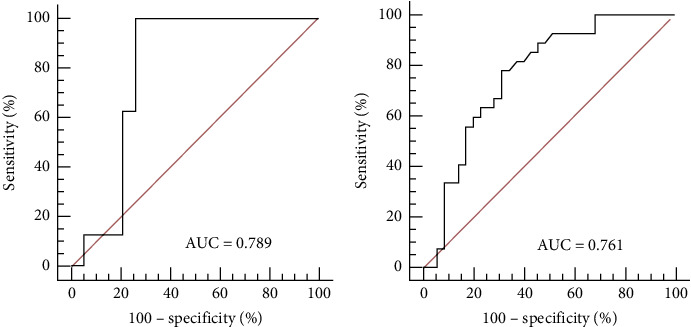
The AUC_ROC_ of proximal MNBI and weakly acid reflux for predicting the efficacy of standard antireflux therapy. (a) When proximal MNBI was used to predict the efficacy of standard antireflux therapy, the AUC was 0.789 (95% confidence interval (CI): 0.590–0.921), with a moderately good predictive ability. When 2140Ω was adopted as a cutoff point, the Youden index was the highest (0.629). (b) When weakly acid reflux was used as a predictor, the AUC was 0.761 (95% CI: 0.636–0.860), with a moderately good predictive ability. When 45 was adopted as a cutoff point, the Youden index was the highest (0.401).

When using the proximal MNBI to predict the efficacy of standard antireflux therapy for nonacid GERC, the AUC_ROC_ was 0.789 (*P*=0.001), and the optimal cutoff value for proximal MNBI was 2140 Ω (Figure 2). Using a proximal MNBI of < 2140 Ω as the predictive indicator for therapeutic efficacy, the AUC_ROC_ was 0.814 (*P*=0.001), with a Youden index of 0.629, positive predictive value of 68.42%, negative predictive value of 93.33%, specificity of 70%, and high sensitivity of 92.86% (Table 4). Using weak acid reflux as a predictor, the AUC_ROC_ was 0.761 (*P* ≤ 0.001), with an optimal cutoff of 45 (Figure 2). When weak acid reflux  > 45 was used as a predictor, the AUC_ROC_ was 0.686 (*P*=0.001) with a Youden index of 0.373, specificity of 62.96%, sensitivity of 74.29%, positive predictive value of 65.20%, and negative predictive value of 72.22% (Table 4).

### 3.5. Predictive Value of Proximal MNBI < 2140 Ω Alone, Weak Acid Reflux > 45 Alone, and Their Combination for Predicting the Efficacy of Standard Antireflux Therapy

The Delong test showed that using MNBI <2140 Ω as a predictor alone had a higher predictive value than weak acid reflux > 45 (AUC_ROC_ = 0.814 vs. AUC_ROC_ = 0.686, *P*=0.029). Furthermore, using the combination of proximal MNBI < 2140 Ω and weakly acid reflux > 45 (AUC_ROC_ = 0.814 vs. AUC_ROC_ = 0.733, *P*=0.577) or using either proximal MNBI < 2140 Ω or weakly acid reflux > 45 (AUC_ROC_ = 0.814 vs. AUC_ROC_ = 0.683, *P*=0.028) did not improve the predictive value. Thus, proximal MNBI < 2140 Ω was the best predictor of standard antireflux therapy efficacy in nonacid GERC patients, with a higher sensitivity and AUC_ROC_ (Table 4).

## 4. Discussion

In this study, nonacid GERC patients who responded to standard antireflux treatment had a lower MNBI and proximal MNBI and more episodes of weakly acid reflux, nonacid reflux, gas reflux, and proximal extent reflux than nonresponders. Multivariate logistic regression revealed that proximal MNBI and weakly acid reflux were independent predictors of standard antireflux therapy. In addition, further analysis combined with the ROC curve analysis showed that the diagnostic value of proximal MNBI < 2140 Ω was higher than that of weak acid reflux > 45.

Nonacid reflux was characterized by the pH value and further separated into weakly acidic (pH 4–7) or weakly alkaline (pH>7) [3]. While most studies have focused on acid GERC, nonacid GERC has also been reported and can sometimes be the crucial aetiology [27–30]. A meta-analysis [28] revealed that in SAP-positive GERC patients in whom acid suppressive therapy was discontinued for 2 weeks, the percentages of acid, weakly acidic, and weakly alkaline reflux were 65%, 29%, and 6%, respectively. In another study [5], 37% of the patients with GERC in whom PPIs had been withdrawn had nonacid GERC. However, in patients undergoing antiacid therapy, nonacid reflux occurred at a higher rate (80%–82.7%) [5, 31]. We previously [29] demonstrated that weakly acid reflux was the main cause of cough symptoms in nonacid GERC [29]. Reflux episodes reaching the proximal oesophagus and the pharynx were observed in nonacid GERC patients [32, 33]. Among them, the incidence of nonacid reflux was fairly high, accounting for 73% in the proximal oesophagus and 11.1% in the pharynx [32].

In recent years, nonacid GERC has been increasingly diagnosed with the use of MII-pH monitoring. However, the treatment of nonacid GERC is still clinically challenging. Neither the chronic cough guidelines [2, 7, 8, 21] nor the recent Lyons Consensus [20] illustrate the effective management of nonacid GERC. Both types of GERC (acid and nonacid) patients were pretreated with standard-dose PPIs for 8 weeks when there was definite abnormal reflux [7, 34]. However, some nonacid GERC patients who did not respond to standard antireflux therapy [8, 34] did respond to subsequent neuromodulators, such as baclofen [3, 10, 11, 13, 14]. For this reason, it is necessary to identify nonacid GERC patients who respond to the standard antireflux treatment early and directly treat these patients with neuromodulators, which can quickly alleviate their cough symptoms, improve their compliance, and avoid unnecessary treatment, thus improving the overall efficacy of nonacid GERC.

This study revealed that proximal MNBI and weakly acid reflux were independent predictors of standard antireflux therapy. During MII-pH monitoring, different media (gas, liquid, and mixture) generated different impedances when passing through the electrode patches at each end of the monitoring catheter, and the MNBI was a stable value when the subjects were at a steady state during the night without a bolus passing through. Prolonged exposure of the oesophageal epithelium to luminal acid increased the permeability of the oesophageal epithelium in a dose-dependent manner, in part by modulating the amount of claudin-1 and claudin-4 [35]. This resulted in decreased transepithelial resistance, increased paracellular permeability, and dilated intercellular spaces (DIS) [36]. Thus, the baseline impedance (MNBI) decreased [36]. Acid exposure correlated with macroscopic and microscopic lesions in oesophageal mucosa. The persistence of weakly acid reflux containing bile acids and/or other gastric juice components may induce or maintain both DIS and oesophageal permeability [37]. Distal oesophageal perfusions provoked changes not only in the “exposed” but also in the more proximal “nonexposed” mucosa. A recent study by Caviglia et al. [38] showed that the DIS in NERD patients may lie in both areas close to the gastroesophageal junction and the more proximal oesophagus, which is less exposed to gastric refluxate. This “spread of DIS from the distal to proximal oesophagus” may be related to mast cell degranulation (histamine release), activation of capsaicin-sensitive afferent neurons, and release of neurokinins, which are involved in oesophageal hypersensitivity. The human oesophageal mucosa is very sensitive to continuous exposure to acidic solutions. Oesophageal perfusion with weakly acidic solutions induced DIS of similar magnitude to that provoked by acidic solutions [39]. Therefore, weakly acidic and acidic solutions are equally capable of causing MNBI reduction.

Previous studies in our department have demonstrated that proximal reflux is mainly caused by weakly acidic reflux [40]. Clinical studies have found that 75% of nonacid reflux reached the upper third of the oesophagus [41], which is consistent with our findings in this study. The responders had more weak acid reflux and proximal reflux than the nonresponders. In addition, studies have shown that the ability of weakly acidic reflux to cause DIS and thus to reduce MNBI is comparable to that of acid reflux [39]. In this study, more weakly acidic reflux reached the proximal oesophagus and caused direct damage to the proximal oesophageal mucosa, resulting in a decrease in the proximal MNBI. Furthermore, we demonstrated that weak acid reflux could be transmitted from the lower oesophagus to the high “nonexposed zone” oesophagus via the aforementioned oesophageal hypersensitivity reflex [38], which in turn causes a further decrease in the proximal MNBI. We suggest that this is the reason why the responders had a lower proximal MNBI than the nonresponders and that the predicted antireflux effect was due to the superposition of the direct stimulation of the proximal weakly acidic reflux and the stimulation of distal reflux through the oesophageal hypersensitive reflex. In this study, nonacid GERC patients with weakly acid reflux > 45 and proximal MNBI < 2140 Ω still had increased acid exposure (weakly acid); thus, treatment with a standard dose of PPIs alone was sufficient to reduce the acidity of the reflux content.

A significant proportion of patients in this study responded to standard antireflux therapy. Combined with previous studies in our department [11–13, 18], the reason why standard antireflux works in some patients with nonacid GERC can be attributed to some of the following reasons: (1) Patients with nonacid GERC were rigorously screened, and other possible causes of cough were excluded according to a step-by-step procedure. Combined with the objective parameters of MII-pH, the diagnosis of nonacid GERC was finally established. (2) Patients were amenable to PPIs and were different from those refractory to a high dose of PPIs reported in previous studies [3]. (3) The role of small amounts of acid reflux cannot be ruled out even though nonacid reflux is the main cause of cough (this can be seen in the results of this study, which showed that patients who responded to standard antireflux had more weak acid refluxes). (4) The slight increase in the pH value of the refluxate caused by PPIs may effectively reduce the stimulation of weakly acid reflux to oesophageal mucosa [3]. (5) Prokinetic drugs promote the emptying of the oesophagus and stomach and reduce the volume of refluxate and frequency of reflux. Oesophageal dysfunction and airway inflammation and hypersensitivity are more likely to be the underlying aetiologies in patients with nonacid GERC who do not respond to standard antireflux treatment. The additional treatment of a muscle relaxant such as baclofen can not only reduce both types of reflux (acid and nonacid) by inhibiting transient lower oesophageal sphincter relaxation but also act as a nonspecific antitussive medication [11].

Although logistic regression showed that both proximal MNBI < 2140 Ω and number of weak acid reflux > 45 could predict the therapeutic effect of standard antireflux therapy, ROC curve analysis indicated a higher predictive value of proximal MNBI. The relatively low sensitivity (62.96%) of weakly acid reflux > 45 as a therapeutic efficacy predictor might cause the majority of patients with nonacid GERC to be omitted. In addition, given the high susceptibility of weakly acid reflux events to be affected by the patient's lifestyle (i.e., the type of food, drugs, and meal), the proximal MNBI demonstrates its relatively stable and reliable characteristics as an objective indicator. Therefore, in future, a proximal MNBI < 2140 Ω can be used to accurately predict the response to standard antireflux therapy in nonacid GERC patients. In addition, weakly acid reflux > 45 can also be used as a suboptimal secondary predictor when MNBI data are not available. These two indicators can be used to target the treatment of nonacid GERC, quickly relieving the cough symptoms of some nonacid GERC patients and achieving an overall improvement in treatment efficiency and accuracy. Notably, a recent study [42] in which suspected GERC patients were stratified according to AET (>6%; between 4% and 6%; and < 4%; erosive oesophagitis was excluded) reported a correlation between pathological MNBI or PSPW index and PPI response. The differences between the results of our study and those mentioned above can be explained by the different study subjects. However, both studies propose that MNBI, a novel MII-pH variable, can make up for the limits of AET and SAP/SI.

The major limitations of this report are the small sample size and recruitment from a single centre. Further verification with a prospective study is needed. However, given that nonacid reflux is not that common in the GERC population, it is difficult to achieve a large sample size. Placebo effects can also not be excluded. However, it is not a clinical drug trial but a retrospective observational study, and remarkable improvement has been reported previously [10–12, 14].

Collectively, standard antireflux therapy should be applied directly to nonacid GERC patients with a proximal MNBI < 2140 Ω. In the absence of MNBI, weakly acid reflux > 45 can be used as an auxiliary indicator. Baclofen is recommended as an additional treatment after standard antireflux therapy.

## Figures and Tables

**Table 1:** Demographic and clinical characteristics of responders and nonresponders.

	Responders (*n* = 50)	Nonresponders (*n* = 40)	*P* value
Gender (male/female)	27/23	20/20	0.775
Age (years)	50.14 ± 12.29	41.27 ± 14.33	0.323
Duration of cough (months)	48.00 (18.00, 89.50)	36.00 (12.00, 84.00)	0.731
GerdQ score	6.00 (6.00, 6.50)	6.00 (6.00, 7.50)	0.801
Cough symptom score			
Daytime	3.50 (3.00, 4.00)	3.00 (3.00, 4.00)	0.596
Nighttime	1.00 (1.00, 2.50)	1.00 (1.00, 2.00)	0.885
Capsaicin cough threshold			
C2 (*μ*mol/L)	0.91 ± 0.22	1.19 ± 0.14	0.622
C5 (*μ*mol/L)	2.63 ± 0.23	2.14 ± 0.15	0.754
Lung function			
FEV1% pred	105.44 ± 9.75	95.17 ± 13.44	0.247
FVC% pred	113.70 ± 11.09	95.42 ± 14.24	0.399
FEV1/FVC (%)	79.99 ± 6.47	84.36 ± 6.63	0.979

Data are presented as the mean ± SD, median (interquartile range), or no. (%) unless otherwise indicated; C2, capsaicin solution concentration for  ≥2 coughs; C5, capsaicin solution concentration for ≥ 5 coughs; GerdQ, gastroesophageal reflux diagnostic questionnaire; FEV1, forced expiratory volume in 1 s; FVC, forced vital capacity.

**Table 2:** Comparison of MII-pH variables between responders and nonresponders.

Item	Responders (*n* = 50)	Nonresponders (*n* = 40)	*P*
AET (%)	1.05 (0.45, 3.00)	1.11 (0.41, 3.38)	0.669
PSWPI	38.68 ± 9.99	50.92 ± 5.39	0.332
MNBI	1817.75 ± 259.26	2369.93 ± 326.35	0.030
Proximal MNBI	1833.39 ± 92.16	2742.57 ± 204.64	≤0.001
Distal MNBI	2095.16 ± 266.62	2778.37 ± 286.62	0.095
SAP (%)	85.80 (73.80, 96.70)	90.35 (65.40, 98.25)	0.508
Acid SAP (%)	20.90 (7.00, 35.58)	24.50 (13.05, 36.05)	0.418
Nonacid SAP (%)	85.50 (65.50, 96.25)	7.00 (0.00, 91.90)	0.101
SI (%)	22.50 (5.00, 35.13)	20.00 (0.00, 33.30)	0.857
Total reflux (*n*)	90.00 (29.00, 148.00)	58.10 (36.20, 91.00)	0.284
Acid reflux (*n*)	20.90 (7.00, 35.58)	24.95 (13.33, 36.63)	0.340
Weakly acid reflux (*n*)	56.00 (31.70, 86.00)	14.00 (14.00, 44.20)	0.022
Weakly alkaline reflux (*n*)	8.75 (1.83, 24.75)	10.15 (1.60, 18.48)	0.907
Nonacid reflux (*n*)	61.35 (15.90.86.50)	21.60 (0.00, 52.50)	0.008
Gas reflux (*n*)	20.00 (10.40, 44.00)	11.10 (4.85, 24.00)	0.059
Liquid reflux (*n*)	20.60 (8.75, 32.00)	17.00 (9.00, 28.80)	0.610
Mixed reflux (*n*)	45.00 (14.90, 67.50)	37.60 (17.40, 56.00)	0.905
Proximal extent (*n*)	19.00 (5.04, 24.00)	5.50 (2.56, 11.13)	0.011
Acid clearance (*s*)	10.50 (7.13, 13.00)	12.00 (10.50, 12.00)	0.307

Note: MNBI, mean nocturnal baseline impedance; PSPWI, postreflux swallow-induced peristaltic wave index; SAP, symptom association probability; SI, symptom index; AET, acid exposure time.

**Table 3:** Multivariate logistic regression analysis of the efficacy of standard antireflux therapy.

Variable	*β*	SE	Wald *χ*^2^	*P*value	OR	*χ * ^2^value	*P*value	*R * ^2^value
Regression model						14.272	0.001	0.422
Proximal MNBI	−0.002	0.001	4.277	0.042	0.997			
Weakly acid reflux	0.039	0.018	4.724	0.029	1.051			

MNBI, mean nocturnal baseline impedance; OR, odds ratio.

**Table 4:** Comparison of proximal MNBI and weakly acid reflux value in predicting the efficacy of standard antireflux therapy.

Standard	Sensitivity (%)	Specificity (%)	Positive predictive value (%)	Negative predictive value (%)	AUC_ROC_	Youden index	Kappa value
*A*	92.86	70.00	68.42	93.33	0.814	0.629	0.597
*B*	62.96	74.29	65.20	72.22	0.686	0.373	0.374
*A* and *B*	50.00	96.55	90.0	75.68	0.733	0.466	0.508
*A* or *B*	84.21	52.38	61.54	78.57	0.683	0.366	0.360
*χ * ^2^	4.885	4.394	11.394	2.814			
*P*	0.001	0.006	0.034	0.032			

*A*, proximal MNBI < 2140 Ω; *B*, weakly acid reflux > 45; MNBI, mean nocturnal baseline impedance; OR, odds ratio.

## Data Availability

The datasets used and/or analysed during the current study are available from the corresponding authors upon request.
